# A glass nanopore ionic sensor for surface charge analysis

**DOI:** 10.1039/d0ra03353g

**Published:** 2020-06-05

**Authors:** Songyue Chen, Hong Chen, Jian Zhang, Hepeng Dong, Kan Zhan, Yongliang Tang

**Affiliations:** Department of Mechanical and Electrical Engineering, Xiamen University Xiamen 361005 China s.chen@xmu.edu.cn; Pen-Tung Sah Institute of Micro-Nano Science and Technology, Xiamen University Xiamen 361005 China; College of Chemistry and Chemical Engineering, Xiamen University Xiamen 361005 China

## Abstract

Surface charge-based nanopore characterization techniques unfold unique properties and provide a powerful platform for a variety of sensing applications. In this paper, we have proposed a nanoconfined inner wall surface charge characterization method with glass nanopores. The glass nanopores were functionalized with DNA aptamers that were designed for mercury (Hg^2+^) ion immobilization by forming thymine–Hg^2+^–thymine structures. The surface charge of the nanopores was modulated by surface chemistry and Hg^2+^ ion concentrations and analysed by combining zeta potential measurements on glass slides and the ionic current rectification ratio of the nanopores. Also, 1 pM Hg^2+^ ions could be detected by the nanopores.

## Abstract 2

Surface charge-based nanopore characterization techniques unfold unique properties and provide a powerful platform for a variety of sensing applications.

## Graphical abstract

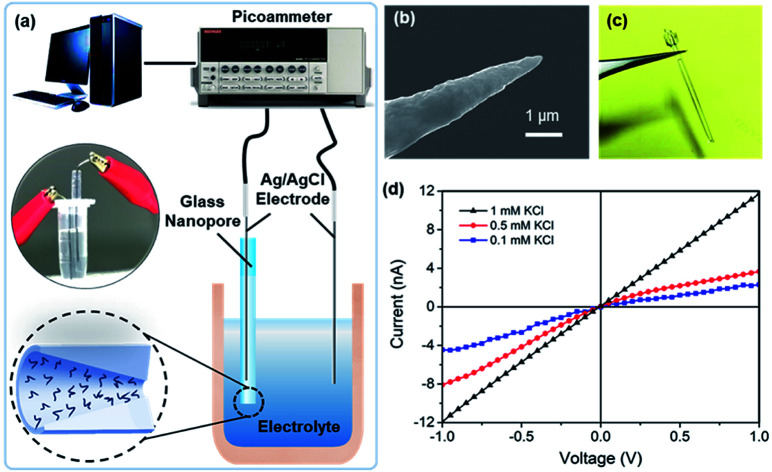

## Introduction

Surface charge-based sensing techniques have attracted tremendous attention over the last decades from ion-sensitive field-effect transistors (ISFETs)^1^ and nanowire field-effect transistors^2,3^ to nanopore sensors.^4–6^ A surface charge sensor monitors changes in the surface charge that are modulated by changing the ionic concentrations or the adsorption of specific molecules. Such devices are sensitive, miniature, capable of being integrated in microfluidic systems and amenable for constructing on-site and portable sensing systems. Among all the sensing materials, nanopores have nano-confined and concave inner wall structures,^7,8^ exhibit unique ion transporting properties, and can be applied in various sensing areas.^4–6,9–11^

The transportation of ions and molecules through nanopores depends heavily on the charge polarity and charge density of the inner wall.^12,13^ Researchers have shown that the surface charge of nanopores can be modulated by pH,^6,14^ metal ions,^4,15^ cation valence,^16^ temperature,^17^ salt gradient, and even the voltage applied across the nanopores.^18^ The non-uniform distribution of the surface charge-induced electric field inside the nanopores leads to ion current rectification.^8^ The rectification ratio is affected by the surface charge, asymmetry of the nanopores, and concentration gradient over the nanopores.^19^ Surface charge sensors can be obtained by designing proper surface chemistry and working conditions. In the case of selective ion sensing, the preparation of recognition sites on the nanopore wall is required, which have been realized with G-quadruplex DNA^4^ and crown compounds^11^ for potassium ion detection, polyglutamic acid^20^ and polyamine-decorated cyclodextrins^21^ for cupric ion detection, and aptamers for thrombin^22^ and lysozyme molecule detection.^5^

In this paper, we studied the surface charge variation at different surface functionalization steps with glass nanopores, and further designed and applied the glass nanopores for mercury(ii) (Hg^2+^) ion detection. The surface charge modulation over the inner wall of the nanopores was studied through the changes in the ionic current rectification of the glass nanopores and further verified with zeta potential measurements on glass slides. Specific thymine (T)-rich aptamers were designed for selective Hg^2+^ ion immobilization by the formation of T–Hg^2+^–T structures. We demonstrated that the changes in surface charge could be monitored for Hg^2+^ ion concentrations as low as 1 pM.

## Materials and methods

### Materials

Phosphate buffered saline (PBS, 0.01 M phosphate buffer, 0.0027 M KCl and 0.137 M NaCl, pH 7.4, at 25 °C), 3-aminopropyltriethoxysilane (APTES, 99%), and glutaraldehyde (GA, 50%) were purchased from Sigma-Aldrich. Mercury binding DNA aptamers were synthesized by Sango Biotech (Shanghai) with an amino group at the 3′ end: 3′-NH_3_-(CH_2_)_9_-TCATG TTTG TTTG TTGG CCCC CCTT CTTT CTTA-5′. HgCl_2_ (99%) was purchased from Huijia Biotech. The remaining acids and salts were all of analytical standard and were purchased from Sinopharm Chemical Reagent. The reagents were prepared in Milli-Q water with a resistance of 18.2 MΩ.

### Circular dichroism (CD) spectroscopy measurements

CD spectra were obtained on a JASCO J-810 CD spectrometer with wavelengths between 220 nm and 320 nm at room temperature. Quartz cells with a path length of 1 mm were used for the testing. DNA was dissolved in a 0.01× PBS buffer solution with a concentration of 5 μM.

### Glass nanopore fabrication

The nanopore fabrication process includes four main steps, which has been reported previously.^23^ The first step was to prepare platinum nanotips by electrochemical etching in a 15% CaCl_2_ solution with an AC voltage applied at a frequency of 64 Hz. Then, a platinum wire was encapsulated in a glass capillary with an alcohol blowtorch. We ground the glass tubes first with sandpaper and then with Al_2_O_3_ powder until the platinum wire was exposed. The last step was the etching of platinum with boiling nitrohydrochloric acid for 4 hours. Then, the glass nanopores were ultrasonically cleaned in DI water and subsequently ethanol. The nanopore radius was determined with an empirical formula.^24^

### Experimental setup

**Fig. 1:**
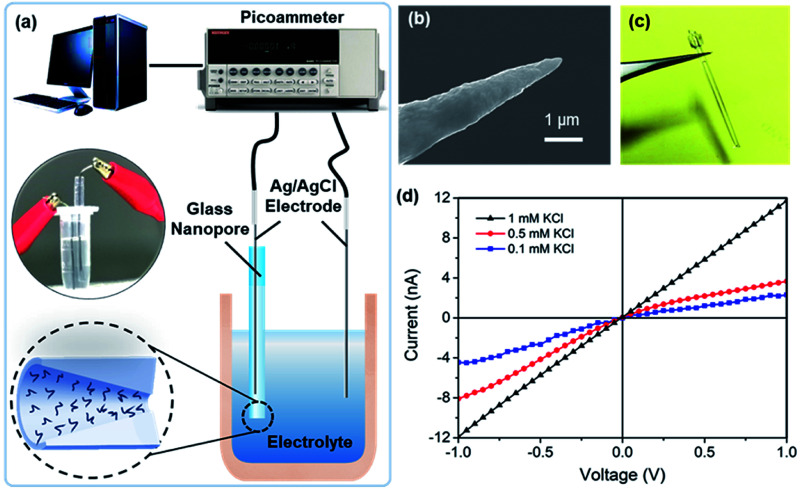
(a) Schematic diagram of a single glass-nanopore setup; (b) SEM image of the Pt nanotip; (c) photo of a glass nanopore; (d) *I*–*V* curves of a nanopore at KCl concentrations of 10 mM, 100 mM, and 1 M and current rectification at lower concentrations.

The glass nanopore detection system is shown in Fig. 1a. Conductance measurements were recorded with a Keithley picoammeter 6487. A 0.01× PBS solution was used as the background solution. All the measurements were obtained in a Faraday cage at room temperature. Each current–voltage (*I*–*V*) curve was repeated three times and average values at different voltages were calculated.

### Surface functionalization

First, the glass nanopores were cleaned with Piranha solution (H_2_SO_4_ : 30% H_2_O_2_, 3 : 1) for 2 h and then washed with DI water and ethanol to obtain hydroxyl groups on the surface. After blowing dry with nitrogen air, the nanopores were immersed in APTES ethanol solution (5% APTES + 2% H_2_O) for 2 h and incubated at 120 °C for 1 h to crosslink silane to the –OH groups. Then, aldehyde groups were introduced to the surface by reacting the amino groups with GA (5% in 10× PBS, pH 7.4) overnight. After cleaning with PBS solution, the nanopores were immersed in 1 μM custom designed DNA aptamer PBS buffer for 1.5 h. The aptamer has an amino group at the 3′ end, which can react with the aldehyde. Finally, the surface was treated with 2.5 mg mL^−1^ sodium borohydride (NaBH_4_) in PBS buffer (pH 7.4) with 25% ethanol for 1 h to chemically reduce the unreacted aldehyde groups.

### Zeta potential measurements

Zeta potential measurements were recorded on glass slides with an Electrokinetic Analyzer (Anton Paar SurPASSTM 3). The glass slides had the same compositions as the glass capillaries used for making the nanopores: 72% SiO_2_, 13.5% Na_2_O + K_2_O, 8.1% CaO, 4% MgO, and 1% Al_2_O_3_. The curves were measured in 1 mM KCl with dropwise addition of 1 mM NaOH or HCl to reach each pH value, *i.e.*, from pH 3 to pH 9.

## Results and discussion

The ionic conductance of a nanopore is a function of the nanopore radius and the surface charge density of its inner wall.^25^ In this study, the diameter of the nanopore tip was chosen to be ∼40 nm. We previously obtained a simplified conductance model for a circular cross-sectioned nanopore filled with an electrolyte with 1 : 1 ratio (in this case, NaCl); this can be expressed as *G* = (*μ*_Na^+^_ + *μ*_Cl^−^_)*c*_o_*N*_A_*e*π*r*^2^/*t* + 2*μ*_Na^+^_*σ*_s_π*r*/*t*, where *μ*_i_ is the mobility of the ion i, *c*_o_ is the concentration of the salt solution, *N*_A_ is the Avogadro constant, *r* and *t* are the radius and length of the nanopore, respectively, *σ*_s_ is the surface charge density, and *e* is the elementary charge. When equal contributions of the dimension and surface charge density to conductance are considered, a charge detection limit *σ*_sl_ = (*μ*_Na^+^_ + *μ*_Cl^−^_)*c*_o_*N*_A_*er*/2*μ*_Na^+^_ ≈ 3.32 mC m^−2^ is expected for a glass nanopore radius of 20 nm and background solution of 1.37 mM NaCl, considering the main composition of 0.01× PBS used during testing. These working conditions correspond to a detection limit of 1 charge per 48 nm^2^.

As indicated by the platinum nanotips used during the fabrication process (Fig. 1b), the glass nanopores are conical in shape. Measurements were obtained with two Ag/AgCl electrodes placed on both sides of the nanopore (Fig. 1c). The *I*–*V* curves of the nanopore at different KCl concentrations (pH 5.7) are shown in Fig. 1d, where the *I*–*V* curve is linear for the 1 mM KCl solution and shows rectification at lower concentrations, *e.g.*, 0.5 mM and 0.1 mM. The conical-shaped nanopores exhibited a current rectification characteristic^25^ due to the non-uniform electric field distribution inside the nanopores. Meanwhile, applying low ion concentrations further enhanced the surface charge effect and improve the charge sensitivity.^26^

**Fig. 2:**
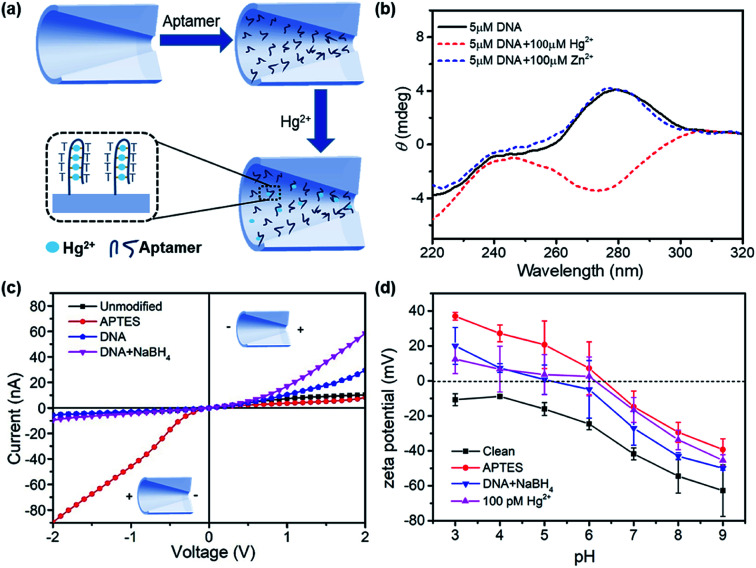
(a) Schematic of the aptamer-functionalized glass nanopore for mercury ion detection (not to scale); (b) CD comparison between DNA and DNA with mercury ion addition; (c) *I*–*V* curves of the glass nanopore for the unmodified, APTES-modified, DNA aptamer-modified, and NaBH_4_-treated surface; (d) zeta potential measurements after different surface treatments in 1 mM KCl with the dropwise addition of 1 mM NaOH or HCl to reach each pH value.

The design of surface chemistry for mercury ion detection is illustrated in Fig. 2a. The DNA aptamer was functionalized on the nanopore, which had six pairing spots for mercury ions. Hg^2+^ ions specifically combined with two thymine bases of the aptamer by forming stable T–Hg^2+^–T structures.^27,28^ This aptamer-based reaction is also selective, reversible, and amenable for multiplexed detection.^29–31^ The conformational change in the aptamer was determined by circular dichroism (CD) spectroscopy, as shown in Fig. 2b. In the absence of Hg^2+^, the DNA solution exhibited a positive peak at 280 nm, indicating a typical conformation of single strand. After adding 100 μM of Hg^2+^ to the aptamer solution, a negative peak near 275 nm appeared, demonstrating the formation of the T–Hg^2+^–T structure.^32^ However, in the presence of 100 μM Zn^2+^, the CD spectrum had almost no change.

Fig. 2c demonstrates the *I*–*V* curves of the glass nanopores after surface treatments: piranha solution cleaning, APTES modification, DNA aptamer functionalization, and NaBH_4_ reduction. The surface treatments had significant impacts on the ionic current as well as the current rectification behavior. The APTES-functionalized nanopore showed a dramatic increase in the current on the left side. Therefore, the current rectification ratio dramatically increased from 2 to 10 but in the reverse rectification direction. This is expected with the positive charge introduction by the amino groups. Then, the rectification ratio reversed again to 6 for the DNA aptamer-functionalized surface due to the negative charge introduced by DNA. After treating with GA, the unreacted –CHO groups were reduced to –OH groups, which introduced more negative charges on the surface. Therefore, an increased current on the right side and higher rectification ratio were observed.

In order to verify the charge polarity of different surfaces, we performed zeta potential measurements on glass slides that had the same composition as the glass capillaries used in the experiments. The glass slides were treated with the same surface chemistry as the glass nanopore. Then, zeta potential was measured in a background solution of 1 mM KCl with dropwise addition of 1 mM NaOH or HCl to reach each pH value. The zeta potential measurements are shown in Fig. 2d. The introduction of amino groups from APTES increased the zeta potential by over 30 mV at different pH conditions. The zeta potential reduced after further immobilizing the DNA aptamers on the surface and rose again with the adsorption of mercury ions. Interestingly, the point-of-zero-charge pH for the APTES surface was ∼6.4, below which the surface is positively charged. However, the *I*–*V* curves of the APTES-functionalized nanopore showed positive charge polarity in the pH 7.4 buffer solution compared with the negatively charged clean surface. Therefore, we expect that the pH inside the nanopore is more acidic than that in the bulk solution;^33^ this was consistent with theoretical calculations,^34^ where a significant deviation of the pH inside the nanopore from that in the bulk solution was inferred.

**Fig. 3:**
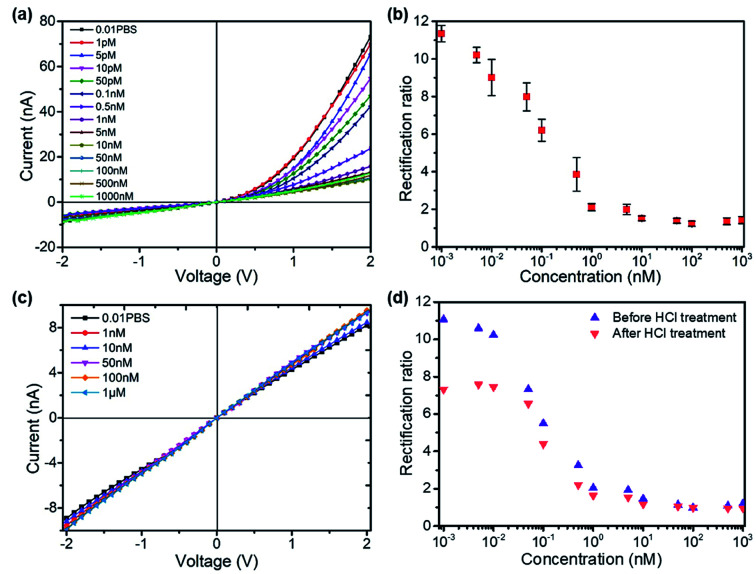
(a) *I*–*V* curves of the functionalized nanopore measuring HgCl_2_ solutions in 0.01× PBS, pH 7.3 with ionic concentrations from 1 pM to 1 μM; (b) the derived rectification ratios from the *I*–*V* measurements, showing decreasing rectification ratios with increasing HgCl_2_ concentrations (three measurements for each concentration); (c) *I*–*V* curves of the bare glass nanopore measuring HgCl_2_ solutions; (d) glass nanopore reusability test for Hg^2+^ sensing after treating with 0.1 M HCl.

The formation of the T–Hg^2+^–T structures in the presence of Hg^2+^ ions further increased the positive surface charge (Fig. 2d). Based on this phenomenon, the functionalized nanopore sensor was used for Hg^2+^ ion detection in a buffer solution of 0.01× PBS. Since each aptamer reacts with a maximum of six Hg^2+^ ions, a larger capacity for surface charge modulation can be expected with the increase in binding sites. The bivalent Hg^2+^ ions form stable bonds with the aptamers.^35,36^ The charge variation on the surface was further studied with the *I*–*V* curves, as illustrated in Fig. 3. The *I*–*V* curves of the nanopores were recorded after immersion in different HgCl_2_ concentrations from 1 pM to 1 μM. We observed reduced ionic conductance on increasing the HgCl_2_ concentration, which saturated at ∼10 nM; over this value, the conductivity increased slightly. Therefore, we expect saturation of the aptamer–Hg^2+^ ion reaction on the surface at high concentrations. The rebounded ionic current is most probably caused by the increased Hg^2+^ ion concentration. The extremely low concentration of 1 pM indicates that there is less than one Hg^2+^ ion in such small space, as calculated from the bulk concentration. Therefore, enrichment of the cations over the nanopores is expected^37^ for such concentrations to be detectable. In contrast, a Hg^2+^ ion detection limit of 8 nM was reported for polymeric nanochannels by immobilizing T-rich ssDNA.^28^ Moreover, a detection limit of 10 pM was reported for glass nanopores functionalized with a macrocyclic dioxotetraamine derivative.^15^ The method reported in this paper has a lower detection limit to date.

Fig. 3b shows the derived rectification ratios of the *I*–*V* curves for each Hg^2+^ concentration. The current rectification ratio is defined as the conductance calculated over the linear region at both positive and negative voltages. We observed decreased rectification ratios on increasing the Hg^2+^ concentration over four concentration ranges, from 1 pM to 10 nM, over which the rectification ratio leveled off at values ∼1; this means that there is no rectification or zero surface charge is reached. The initially negatively charged aptamer surface was neutralized when Hg^2+^ ion adsorption occurred. Therefore, the rectification ratio was proportional to the amount of negative surface charge and eventually Hg^2+^ concentrations over a certain range. Fig. 3c shows the blank tests of bare glass nanopores measured in HgCl_2_ solutions from 1 nM to 1 μM, where a slight increase in conductivity appears due to the increased ionic concentrations. The glass nanopore sensor can be reused after treating with 0.1 M HCl solution to detach the Hg^2+^ ions from the aptamer. The sensor still works for the same HgCl_2_ concentration range despite a drop in the rectification ratio (Fig. 3d). The rectification ratio drop can be caused by the incomplete reaction between HCl and the aptamers inside the nanopores. Alternative sensor recovery could be conducted by adding cysteine, which could remove Hg^2+^ ions from the T–Hg^2+^–T complexes.^28^ Surprisingly, the experiments on Zn^2+^, Pd^2+^, and Cu^2+^ ions did not show clear specificity for the sensors, while the rectification ratio dropped to values between 1 and 2 even at the concentration of 1 pM. A possible reason for this can be the charge attraction between the positively charged metal ions and the negatively charged inner wall. Nevertheless, the present method provides a sensitive way for mercury ion detection at extremely low concentrations. Further optimization is required in order to obtain better performance.

## Conclusions

In conclusion, we analysed the surface charge changes regulated by surface chemistry and ion adsorption with single conical glass nanopores and applied them for mercury ion detection. Zeta potential and ionic current measurements were obtained to evaluate the variation in surface charge with surface modifications on glass slides and glass nanopores. We found a significant deviation of the pH inside the nanopores from that in the bulk solution by comparing both measurements. Both the ionic current and rectification ratio decreased at increased HgCl_2_ concentrations, which resulted from the neutralization of the negative charge of the aptamer-functionalized inner wall. The DNA aptamer-functionalized glass nanopore sensor was very sensitive to Hg^2+^ ions with a detection limit of 1 pM. This study is of great significance for understanding the mechanism of surface charge-based nanopore sensing and for the application of metal ion detection for water and food safety.

## Conflicts of interest

There are no conflicts to declare.

## Supplementary Material
